# Refusals and rituals in sport: photo-elicitation of global Black girls’ and women's joy and care

**DOI:** 10.3389/fspor.2026.1921020

**Published:** 2026-09-18

**Authors:** Asha Edwin, Janelle Joseph, Treisha Hylton, Asma Khalil

**Affiliations:** 1 IDEAS Research Lab, Faculty of Applied Health Sciences, Brock University, St. Catharines, ON, Canada; 2 School of Social Work, Faculty of Liberal Arts and Professional Studies, York University, Toronto, ON, Canada

**Keywords:** Black women, health, leisure, photo elicitation, sport, wellbeing

## Abstract

**Introduction:**

This paper presents four stories of Black women and girls taking up space in sport across global contexts, including Canada, Oman, Brazil, and Jamaica.

**Methods:**

Drawing on Black women's epistemologies and arts-based methodologies, we use collaborative storytelling and photo-elicitation to illustrate how Black women and girls move within and across sporting spaces, producing connections that reflect both shared global histories and distinct local experiences, a hallmark of Black diasporas.

**Results:**

Rather than positioning Black women's sporting experiences solely through narratives of exclusion and harm, this work foregrounds the generative practices through which joy, care, community, and meaning are created.

**Discussion:**

Through four stories and images, we challenge singular narratives of Blackness and instead highlight multiplicity as central to Black life. Black women's forms of ritual and refusal resist racialized and gendered constraints while asserting presence, self-definition, and alternative ways of being. This paper does not only tell, it shows how storytelling through visual inquiry opens space for readers to engage with Black women's experiences and how Black women athletes sustain intergenerational knowledge, belonging, and identity. We expand arts-based approaches in sport studies, extend visual storytelling methods, and advance understanding of global Black identity as lived through movement, connection, and multiplicity.

## Introduction

Guided by Black women epistemologies and arts-based inquiry, we explore how Black women's sporting experiences expand what is known within and beyond sport studies. We centre Black women in sport and story how Black girls and women contribute to, shape, and transform what is known about us within and beyond sport. The problems this paper addresses are threefold. First, there is insufficient representation of Black women within the sociology of sport. Black women athletes thrive in recreational, amateur, and competitive settings, yet their experiences remain underexplored relative to other groups (1, 2). We contribute to a small but growing body of research on Black women in sport, including Joseph et al. (3), Carter-Francique and Richardson (4), and Razack et al. (5), extending the literature by demonstrating the crucial influence of Black women's sport stories and imagery as sites for rich, critical conversations about how identity, care, and wellbeing take shape through Black women's participation in sport.

Second, much of the existing work documents misogynoir (6) and exclusion (4), but far less captures joy, being, and everyday life. This gap reflects a larger pattern in which Black sportswomen have been represented rather than known, in media as well as sport scholarship. Razack and Joseph's (6) analysis of the 2018 women's US Open final, for example, shows how sport media actively articulates and reinforces negative tropes about Black women. Naomi Osaka's Blackness was erased through an emphasis on her Japanese nationality, while Serena Williams was cast through caricature as an “angry, Black force of nature” [(6), p.298], reading Black women through anger rather than joy. Centring joy and being, then, is more than just a corrective addition to literature but a necessary intervention into how Black women's sporting lives get told.

Third, arts-based inquiry remains marginalized within sociological scholarship, despite its importance as a way of documenting and understanding lived experience (7). Within Black studies, art is understood as a critical means of extending the archive, documenting Black livingness (8), practicing refusal as a form of worldmaking through images (9, 10), and producing creative counter-narratives that open both imaginative and lived possibilities (11–13). Together, these problems are addressed through in ‘memory work,’ which Ohito (14) describes as essential to understanding how the present makes claims on Black women's personal, collective, and historical pasts – especially those erased by dominant archives – as both a gesture toward the certainty of the past and a confirmation of the present. This paper offers a Black diaspora feminist arts-based method of inquiry into global sporting Blackness.

Building on multiple storytelling traditions mobilized in sport studies (15–18), this paper emphasizes a unique methodo\logical process among four Black women researchers, each sharing stories about Black women athletes. Through a collaborative storying and photo-elicitation exercise, we generated narratives rooted in African, Black, Caribbean, and Islamic feminist epistemologies. Black diasporas have been theorized in terms of their diversity and similarities since the early 1990s, with particular attention to women's experiences (8, 13, 19, 20) What is clear from this literature is that Black women in the diaspora, whether they are immigrants or their ancestors arrived decades or centuries ago, share identities and conditions “grounded in intersectionality – of race, ethnicity, gender, class, sexuality, age, and nationality –and in understanding the effects of multiple systems of oppression and privilege on their lives” [(19), p. 30]. Black women's narratives are essential to what Collins (11) and Nadar (21) identify as alternative knowledges that affirm ways of being, reject dominant claims to objectivity, and work toward their transformation. Black diaspora women's narratives have shown them to be central to community revitalization, critiques of dominant social structures, and enabling understanding of their multiple positionalities. What has been explored in limited fashion is the role of sport in making our diasporic, trans-, and multi-national lives. Joseph [(16), p. 52] advises, “as physical cultural studies scholars, we must use stories to make meaning of, interpret, and/or (re-) present data. We must find ways to translate, explicate, document, tell and show stories of participants’ physical cultures” if we are to write ourselves into a discipline that has subordinated and marginalized our lived experiences.

Our attention focused on running in Toronto, Canada; weightlifting in the Sultanate of Oman; capoeira, a Brazilian martial art; and surfing in Bull Bay, Jamaica, to explore the relationships that form among Afro-Brasileiras, Afro-Omani Muslim women, Black Jamaicans, and Black Canadians in sport. Through this process, we demonstrate how Black women and girls actively create and reclaim space and experience, respond to, and resist oppression. Our reflections and photo-elicitation conversations explore how Black women and girls experience race and gender through sport; as refusal and ritual; how care and femininity are deeply connected to care for self and others through embodied sporting practices; and how understandings of Blackness emerge through engagement with global Black sporting experiences.

The lingering consequences of colonial and imperialist formations continue to reveal the subjugation of Black women's bodies within and beyond sporting spaces and identities. Drawing on Fuentes (22) and McKittrick (8), we understand archival power and white supremacy as forces that continue to control dominant narratives and official histories about Black women, an authority we actively refuse. As Lobo (23) argues, archives not only play a significant role in preserving collective memory and producing history; they can also resist and challenge dominant assumptions, epistemic violence, and power within archival practices. Importantly, archives and narratives surrounding Black women's gender, sexuality, and physicality supports how Black women see and imagine the self and the futures that are right here and now, honouring those who came before us and will come after us. Drawing on the work of Patterson and colleagues (24) on Black feminist thought as methodology, we detail below the epistemic, theoretical, and methodological perspectives that inform this paper. We then move into an illustration and interactive application of Black feminist theorizing and methodology as memory work, grounded in storytelling and responses to questions about four images.

## Collective visual storying as a Black feminist writing methodology

Methodologically, the work sits at the intersection of photo-elicitation and visual inquiry. This body of scholarship refuses the photograph as a transparent record of reality, treating it instead as a generative prompt and a medium of communication between researcher and participant that does not carry fixed meaning until it is taken up and worked through (25, 26). As Harper argues in his foundational account, the photo-elicitation interview is not simply a process that “elicits more information,” but rather one that “evokes a different kind of information” [(27), p. 13]. Read alongside the wider field of visual methodologies, which holds that images do not merely illustrate but actively create knowledge and surface the tacit and the unspoken (28), this approach allows us to understand each photograph as a site where meaning is made and co-created. Images produce what Spiegel (29) refers to as “visual knowledge” that often goes unexplored, asking what each photograph might teach us about ourselves, sport, and identity and what our research background helps us “see” in terms of history, geopolitical context, and embodied potential. We did not treat the photographs as data to be coded, sorted, or made legible to an external analytic frame. Instead, we treated them as sites of theory and as embodied archives, repositories of gesture, relation, memory, and refusal that hold forms of knowledge the sociology of sport routinely fails to register. We draw on Spiegel's (29) call for greater attention to “women's place-based storytelling and its communicative power, highlighting the significance of positionality when studying socioecological visualization, affect, and change” (p. 120). More specifically, we draw on Jennifer C. Nash's [(30), p. 6] notion of beautiful writing, which allows us as writers to stay “closer to a truth of loss” and, in the context of this paper, close to the images as a way to attend to the affective, emotive, and felt. Following McMahon's (31) attention to slow, repetitive, and imaginative practices, we engage techniques of sitting with images, that is, giving sustained, reflective attention to visual bodily and environmental details, which allow us to interrupt dominant imperialist and colonial ideas about Black women's bodies and experiences while reimagining geographies, temporalities, and meanings, particularly in sport. In exchange, we invite the reader into intimate dialogue as a tool for undoing, decoding, and visioning the social worlds of Black women and Black identities, individually and globally. In doing so, this paper offers a unique contribution to both arts-based research and sport scholarship through its attention to Black women, an understudied population, and dialogic, collaborative, reflection activities. Through this grounding, we co-theorize across the images, reading them alongside one another for what they reveal about Black women and girls’ identities, bodies, environments, and health as lived in and through sport in global contexts.

Shaping how we looked at the images was a Black feminist practice that attunes us to what is visible and not visible, calling for ways of seeing and listening to images influenced by scholars such as bell hooks, Audre Lorde, Tina Campt, and Saidiya Hartman. Their work opens the lenses through which we learned to read our images through refusal and care, opacity, ritual, and world-making. From Campt, we learned a way of looking that “listens” for what an image holds beneath its surface, attending to the quiet frequencies of refusal through which a photograph resists “the very terms of photographic subjection [it was] engineered to produce” [(9), p. 5]. From Hartman, we take up a discipline of restraint and a commitment to sitting with the opacity of what an image may hide or reveal, grounded in a “refusal to fill in the gaps and provide closure” [(12), p. 12]. These stances shape not only what we look for, but how we look, attending to what resists capture and familiar interpretation.

## Locating ourselves and methods

We came together as four independent Black women scholars and researchers, one PhD candidate, one Postdoctoral fellow, one Assistant, and one Associate professor, through overlapping connections, networks, and mentorship. We each identify with Canada, Oman, Jamaica, and Brazil through our personal heritage, research sites, or residence. Some of us view ourselves as settlers and others as visitors, carrying connections to elsewhere while wrestling with our relationships to here, Canada, where we all currently reside. In different and sometimes overlapping ways, we take up both positions as we move in and out of these four nations, which shapes how we understand and create a sense of a shared Black global identity as four Black diaspora women.

Our method of collective visual storying merged with participatory photography. Over 30 weeks, we gathered virtually to impart details from our sport and research experiences across four different continents. The idea to share images emerged organically from our discussions of Black global identity and sport. We each had been moved emotionally, and inspired to move physically, by images of Black women athletes in varied global settings. We each had a strong desire to share these images to guide discussion about how we understand ideas of Black women's global identities. We engaged these images through a praxis of reading together as a way of sensemaking and imagining collective narratives that build on embodied experiences while creating a living archive of Black women's experiences (32, 33).

Over three phases of a facilitated, structured writing exercise, we practiced attuning ourselves. Each meeting lasted between one to two hours, where we began by checking-in with each other personally as part of our Black women's epistemology that flattens hierarchies, centers researchers as whole beings, honours positionality and the multiple lived realities/vulnerabilities we each bring to meetings and builds safety and trust. We then moved into a reflective exercise prompted by guiding questions that involved writing and then discussing our reflections, each time building on earlier conversations. In phase one, attunement, we began with a practice of sharing images we had selected independently and wrote in response to a single question: what is happening here beyond what is immediately visible? Because the images came from separate sites (public websites, social media, and research projects) with different amounts of visual material, we did not view other photographs nor contribute to the selection of each other's image. Instead, we described what we saw in and beyond our own images and then invited each co-author to reflect on each picture with prompts to attend to the body, posture, space, movement, stillness, tension, relation, objects, and light, refusing the pull toward quick interpretation in favour of slower, embodied engagement.

In our second phase, a practice of image storying, we were moved to ask, what does this moment hold that would otherwise go unseen? W traced what (we imagined) unfolded before and after the captured moment, attending to the relationships and practices embedded in the frame, the forms of care and ritual present, the negotiations that may have shaped the moment of capture, and the reasons each image had stayed with the person who selected it. Figures 1–4.

**Figure 1:**
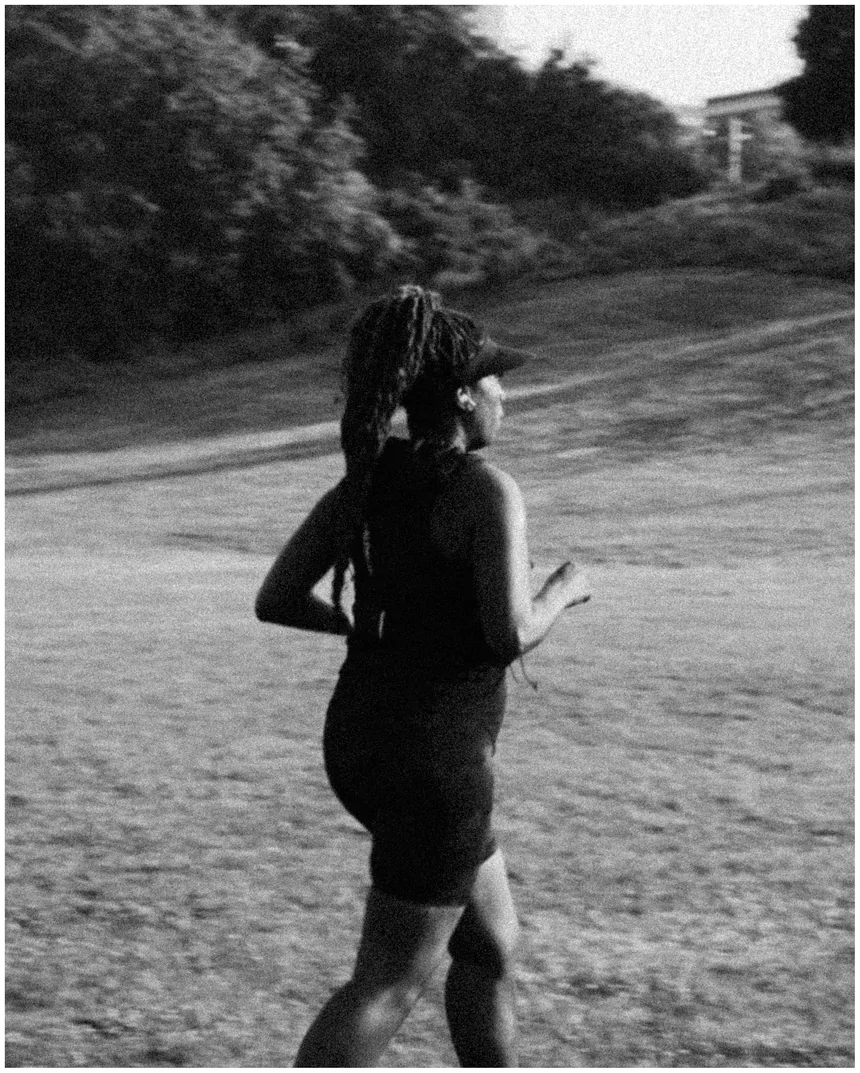
Reprinted with permission from The Body is Home Community Research Project by Chrris Lowe, July 13, 2024.

**Figure 2:**
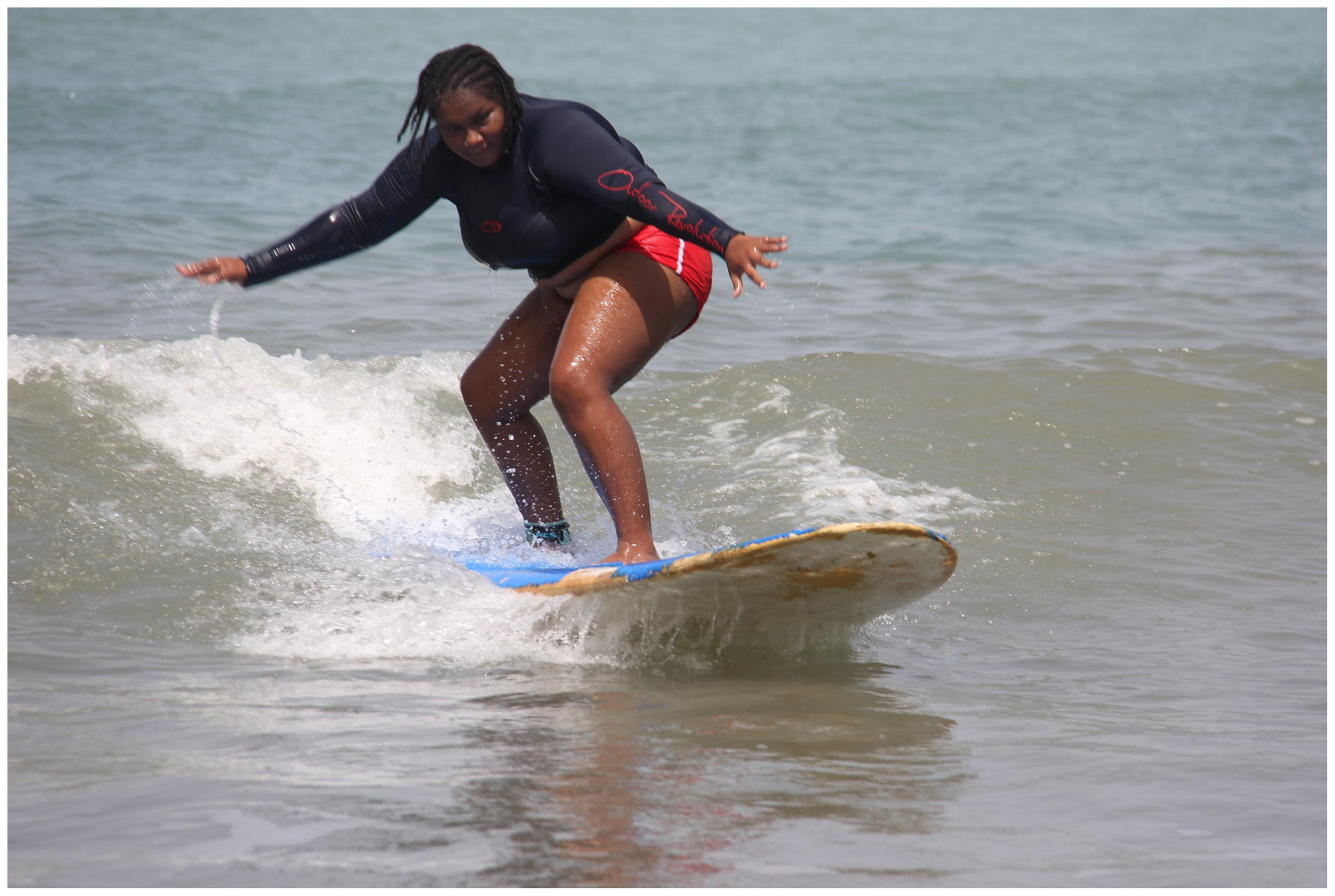
Reprinted with permission from Surfing by Amika, July 10, 2023.

**Figure 3:**
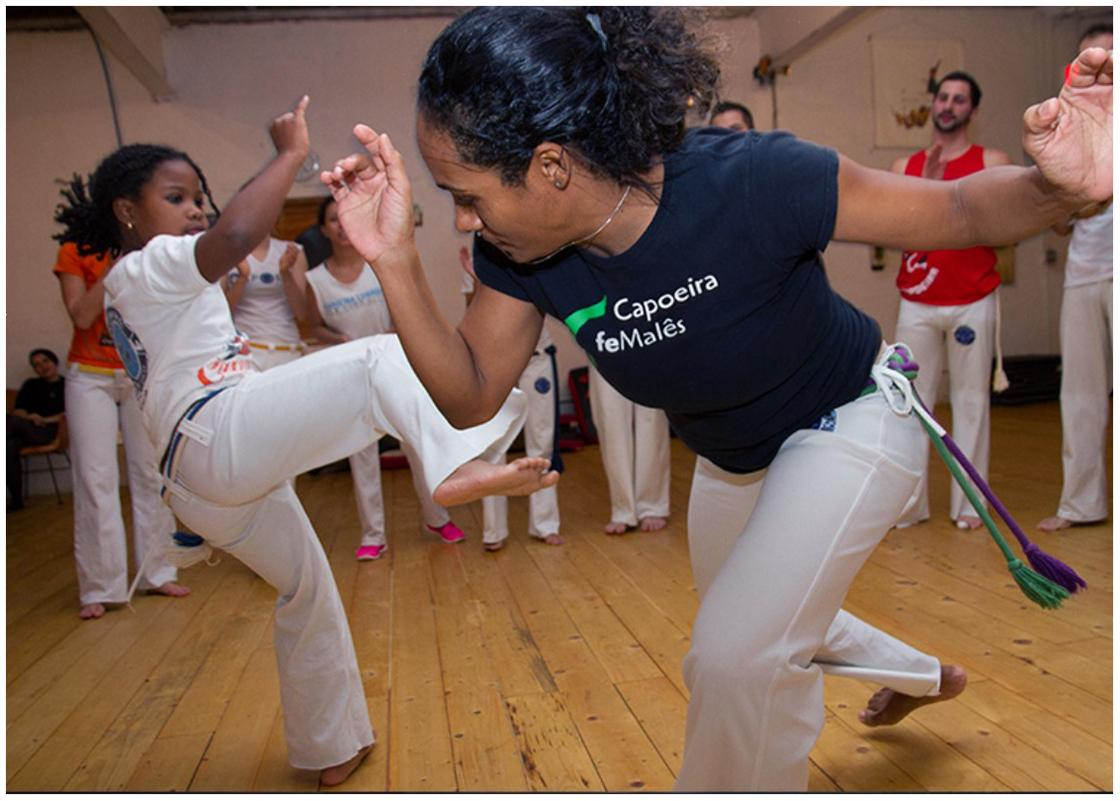
Reprinted with permission from Capoeira by Curtis Peters, July 13, 2016. Available from: https://www.capoeiraschool.ca/

**Figure 4:**
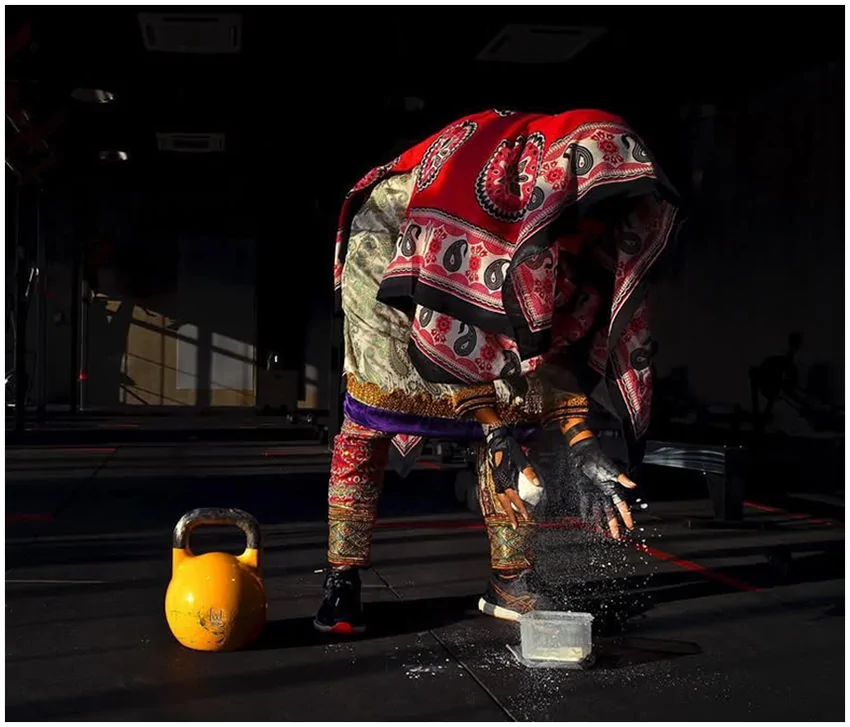
Reprinted with permission from HUN'na by Muna Khalfan, March 08, 2021. Available from https://www.instagram.com/p/CMKKloSjhY6

Sources clockwise from left: Running photographed by Chrris Lowe; Capoeira photographed by Curtis Peters, Surfing photographed by Amika, Kettlebell photograph by Noor alHuda al-Munthari.

## Seeing ourselves – reading images

### Run as one

The selected photograph captures a Black woman running through an open field in downtown Toronto, Ontario, during marathon training season. The runner is one of many Black women who move in community as members of Hill Studio Wellness, an intergenerational Black women's running and wellness club founded by Allison Hill. Thinking back to being in that space, I cannot separate the embodied, felt experience from my understanding of the image or from my personal experience as part of the running group that day. Training often began early in the morning, and I remember the excitement and anticipation of that particular session set against the quiet streets of Toronto before the city filled with people. As the women arrived, the gentle sunrise and early-morning fog welcomed us, and by mid-morning, the sun's warmth touched our skin. This is how I read the image: in proximity. By this, I mean a closeness to my identity, experiences, and sensations in conversation with what I am seeing. The picture moves me into conversation beyond the image, to also being in dialogue with myself. In listening with, in, and through the image alongside my personal knowing, I experience both its obscurity and its depth through aesthetic opacity, yet also its clarity through a woman's moving body.

In its presentation, monochromacy, and grainy texture, the image exposes intricacies of identity and Black womanhood, revealing how these converge in ways that often go unnoticed in the moment. It shows and simultaneously obscures how one exists, moves through, and navigates the world. Against the vastness of nature, where the open field meets the sky almost seamlessly, a woman comes into view. She remains largely faceless, aside from the faint outline of her side profile. The image invites both distance and familiarity, the beauty of the unknown. It resonates as both a felt and metaphorical expression of movement, of going from and toward, with and through something, whether that is the intensity of a high-impact workout, the thoughts we tell ourselves during adversity, the anticipation of what comes next, or the relief of making it to the end of training, of a race, or of the hill. In seeing the image, I also see myself. I witness the runner and glimpse my own movement practice, which flashes through my mind, leaving a lingering feeling and prompting reflection on being in that moment and where I am presently.

It reminds me that the course one takes is never solely black and white, nor is it ever a straight line. There are pivots, curveballs, conditions, and so much colour that make their way into our worlds, filling different spaces and moments in our lives. There are also texture, turbulence, and tenacity, all of which shape how tightly we hold onto our breath, an idea, or a moment, while also shaping what it means to feel our way through discomfort and loosen our grip. There is flux, there is flow, there is freedom, reflecting a shared experience that acknowledges the depth of Black womanhood, embodiment, and interiority through everyday practices of movement. There is a sense of loss, but there is also life. In its simplicity, the image suggests that moving through space is itself a form of presence and agency, a living one, and that Black women quietly resist, reclaim, remake, and reimagine self, space, and sport.

## Chalk and kanga

The photograph is dark at first glance. A woman bends over a chalk bucket in the middle of a gym, her face obscured beneath layers of patterned fabric, her body folded inward and suspended in the particular stillness that precedes motion, the moment before the body commits itself to what it has already decided. Light cuts across the room in a sharp diagonal, catching the chalk dust as it rises from her hands and illuminating the yellow kettlebell resting beside her. What the image captures, in this suspension, is *niyyah*: the Islamic concept of interior orientation, the intention that must precede action and without which the action itself carries no weight. This is that moment, held.

She wears traditional Omani sirwāl, but layered over top is a Swahili kanga, the fabric draping across her back and shoulders as she reaches toward the chalk. It is this layering that immediately invokes Indian Ocean worlds and Afro-Arab histories so often rendered absent from accounts of both the Sultanate of Oman and Blackness. The Swahili coast, Zanzibari communities, the long genealogies of kinship, trade, migration, empire, and displacement through which the Gulf was made are felt. To wear the kanga in the gym is to carry those histories into a space not usually legible as Black, Arab, and Muslim women's home, a provocation that unsettles assumptions about who belongs within sporting spaces as it unsettles who belongs within the geographies of Black identity itself.

This image comes from Hun'na, a community platform and visual archive centering female athletes and adventurers in the Sultanate of Oman, committed to documenting women in movement outside the aesthetic and narrative conventions through which Arab and Muslim women athletes are typically framed. The archive itself must be understood as an act of refusal, a form of self-documentation that asserts the right to exist in the record on one's own terms. The woman in this image is not a placeless or abstracted diasporic subject. She is embedded in a specific ecology, a specific genealogy, a specific sensory world. Chalk on her hands, Swahili fabric against her skin, her body oriented entirely inward, toward herself and toward what she is about to do, in a space that a community built so that this image could exist within it.

I had been staring at this image for a while, sitting with it, thinking through the different ways of looking that it seemed to demand. As I raised the brightness, I noticed that the face was not completely obscured. At the edges, just visible, is the outline of a smile. That the image holds this, that it requires something from the viewer before it presents itself, feels significant. Here, the smile does not perform for the camera but simply exists, waiting for the kind of looking that is slow enough, careful enough to find it.

## Capoeira and community

The photo represents key members of a martial arts group in action. In the foreground, a woman and her daughter play capoeira, a martial art-dance-game of Afro-Brazilian origins. Like many traditional African dances, the conventional capoeira formation represented here involves a group of people standing in a circle, a *roda* (pronouced ho-da). In the photograph, the group supports two of their most senior and most junior players with instrumental, vocal and percussive music to encourage the players to show a *jogo bonito* (beautiful game), propelling their bodies around the *roda* in synchronicity. The room is electric with a cacophony of cheers, music, panting of breath and squeaking of floors, which cannot be heard but must be imagined alongside viewing this photo. Important here is the dialogue between this Black girl and mother, centered in the image and in the community. The entire group shows up to support and empower them, and they simply cannot “do” capoeira on their own.

A *jogo bonito* has the joint connotations of competition and cooperation between two Afro-Brasileiras in a context of mothering, nurturing, learning, and an ethic and ritual of care. The postures of the woman and daughter, whose bare feet contrast with white uniform pants and solid brown hardwood floors, exhibit preparation for attack and defense. To engage in any offensive or defensive technique the capoeirista must *ginga* (sway) to the rhythm created by the entire *roda*, finding the right time to have an embodied conversation. One player might ask, “how are you?” with a kick that sails towards the others’ head and their partner-opponent can answer “I feel amazing today, thank you” by dodging out of the way and patiently preparing a counter-attack with a smile. The ritual of care for self and other exhibited when practicing here reveal kicks are not delivered to hurt, only to show what one is capable of. In the image, the players are far apart enough to protect each other's safety, yet they are engaged in a dance ritual.

Within capoeira, as within life, attack and defense, preparation and action, are always happening simultaneously. Firm lessons, learned through the rituals of capoeira, are to always be prepared, to refuse the status quo, and to avoid collision when evasion is possible. The mother here is being evasive, potentially setting up her daughter for a *rastera* (sweep technique to take out the child's standing leg). Ultimately, this photo captures a pedagogical moment of how to stay safe. This is a martial art that is passed down intergenerationally from teachers to students, mentors to apprentices, mothers to daughters – an essential message for Black livingness.

The image evokes patience and playfulness. They are suspended in air, held by the community, and their ancestors. The group name, Capoeira Malês, harkens back to a group of Muslim enslaved and free African men in Brazil in the 1800s. Their religious and racial revolt in the northeast of Brazil in Salvador, Bahia, is legendary in this region, known as the locus of Afro-Brazilian culture and the origin site of capoeira. The Malês uprising, the largest ever urban slave revolt in Brazil took place on January 24, 1835, and ended in a massacre among the enslaved; however, it remains represented in this image as a lesson about the inherent power of the underclass, the way an opponent can generate a surprise attack at any time, and the potential of the small to overcome — or at least engage on equal footing — with the mighty. Malês is represented here in a playfulness that extends to their sartorial choices. The child is in a traditional white uniform while the mother wears black shirt with the word “feMalês” emblazoned across the chest, making a play with the English word female and the word Malês, used to refer to the multi-ethnic rebel group. Women were indeed a small minority of those involved in the revolt and a small minority of capoeiristas in Brazil until recently. Yet, they were/are there. This photo is proof.

## We are ocean people

For me, as the researcher, choosing this image was intentional. I have hundreds of photos from a two-week ethnography and photovoice project on the intersectional experiences of Black girls surfing in Jamaica, yet this image stood out. Conducting research with Black girls, whose experiences are often extracted from rather than invested in, requires an ethics of care and accountability. I have attempted to resist extractive practices, while recognizing that research is inevitably entangled in forms of extraction. These tensions and contradictions remain part of an ongoing process of reflection.

The Black girl in this image disrupts dominant narratives and aesthetics of surfing. As a larger-bodied Black girl in the ocean, her presence challenges the white gaze that shapes who is imagined to belong in ocean sports. Her body reads as powerful, skilled, and joyful. This is significant not only because she is creating alternative ways of being in surfing, but also because she is new to the sport. I want audiences to see the possibilities of Black girlhood and to witness the multiple ways in which we exist. In her, I see myself, not because our lives mirror one another, although they might, but because the image creates a space of recognition. It suggests that it could be me, and that is enough to create a meaningful connection.

In Jamaica, only about 1 percent of beaches remain accessible to the public due to ongoing privatization, reflecting a broader neocolonial context of dispossession. Bull Bay, where this project took place, is one of the remaining public beaches, yet it is under threat from resort development. Archiving this image becomes, in part, an act of refusal in itself, a way of asserting that we, as Jamaicans, refuse to be erased from our own land and instead reclaim our sovereign relationship with the ocean. Sporting practices such as surfing take on additional political significance if one chooses to look a little longer. Black girls’ presence in the ocean not as tourists but as locals building their skills and relationships, become examples of refusal and reclamation. They assert a right to land, sea, movement, and joy in spaces shaped by colonial histories and ongoing dispossession. These practices move with Black women and girls across land and water, across spaces shaped by constraint and possibility.

## Discussion: storying Black women’s lives through movement and images

Black people have ushered in placemaking, mythmaking, and meaning-making practices rooted in shared and sacred public space, often without being fully aware of their African origins (34). The intervening of past and present, of what is lost and found, becomes a form of resistance aligned with the traditions enslaved Africans carried across the Atlantic, specifically, the connections to water and the deep connection to the African diaspora (35, 36). Colonialism worked to confine African people to representation as a homogenous group of people, largely without diversity and cultural depth (37–39). However, Africa is the most diverse continent, with myriad languages, cultures, ethnicities, knowledge, and practices now spread across the diaspora (40–42). Sport is one practice that deserves attention. The images here represent this diversity, engendering a view of Blackness through multiple lenses affirming that we are diverse, that we are multi-linguistic, and that we exist and belong everywhere globally.

Coupled with this richness in diversity is the shared global history of gendered colonialism and violence. Across global contexts, Black women encounter intersecting forms of racial and gendered discrimination, giving rise to both shared and differentiated lived experiences (43–45). As Lorde (46) notes, “unity implies the coming together of elements which are, to begin with, varied and diverse” (p. 136); or in other words, while marked by diversity, these experiences reflect common structural conditions, building alliances within and across difference. Such histories and connections reveal deep spiritual, familial, collective, and embodied realities that are shared across time and space, contributing to a shared public identity in a global context. Through these connections, Black people engage memory and myth to shape meaning and place while navigating public and private spaces. Through image storying, collective re-membering, we not only ask what has been lost in transition or translation, but we do the work of naming and remembering what remains through shared practices, archival documentation, and collective memory, which are critical for understanding Blackness and Black identity.

In conversation with Ohito's (14) work on revisiting “old” data, we then move into an illustration and interactive application of Black feminist theorizing and methodology as memory work, grounded in storytelling about and responses to four images. We also introduce Harrison's (47) argument that while photographs often or traditionally supplement ethnographic research, images themselves also produce narrative. Building on our reading of images and McMahon's (31) engagement with Saidiya Hartman's practice of reading against the grain, we invite readers to consider potential counter-narratives of the Black women's subjective experiences presented here. Through this exercise and paper, we attest that images operate as narratives and counter-narratives through the authors’ explorations and writings and through the reader's own lens and perspective as a way of seeing, rather than solely through the author.

Engaging with the growing body of research on Black women in sport, including Brown (1), Joseph et al. (3), Carter-Francique and Richardson (4), Razack et al. (5), and Razack and Joseph (6), this paper extends the literature by demonstrating the crucial influence of Black women's sport stories and imagery as sites for rich, critical conversations about how identity, care, and wellbeing take shape through Black women's participation in sport. Additionally, we reveal questions of the archive and its power to protect as well as promote identity, considering where archives live, how they are accessed, and how they take shape through our practices of retaining, selecting, and sharing images. While we have explained what the images mean to us, we also recognize that through the practice of image storying, those who engage with the images may read other narratives onto them and reflect back meanings shaped by their own work and lives.

By critiquing dominant sport spaces and proposing ways to learn from and through Black women's varied sporting experiences, we draw attention to how ideas of Black womanhood, bodies, femininity, and aesthetics are controlled, regulated, and performed. By engaging techniques of sitting with images, we bring together methodological approaches that position the visual and the embodied as central to knowledge production, alongside individual and collective sensemaking that grounds this study. Interrupting dominant narratives of Black women in sport, our analysis and storytelling around each image demonstrate acts of ritual and refusal, understood as embodied practices that sustain memory, connection, and self-definition, while challenging viewers to imagine the possibilities and limitations that exceed imagery itself, including archives and narratives surrounding Black women's gender, sexuality, and physicality. Importantly, this work supports how Black women see and imagine themselves and the futures that are right here and now, honouring those who came before us and will come after us.

## Refusal

Refusal is bound up with ideas of fugitivity and freedom. Sharing an intricate relationship with colonization, particularly through histories of forced movement across water and land, including the Transatlantic Slave Trade and ongoing forms of displacement, refusal has been a key facet of Black life across Brazil, Canada, Jamaica, and Oman and within Black communities globally, necessitated by historic and present legacies of anti-Black erasure, silencing, and violence. From revolts to the creation of new cultures of freedom, these individual and collective practices have produced shared forms of survival, joy, community, and care that move with Black people across land, water, and shifting social and political environments. In these movements, Black people, and particularly Black women and girls, continue to refuse the conditions imposed upon them. What emerges through these histories is a set of imaginaries of otherwise, rooted in joy and sustained through movement, kinship, and collective living.

Across the sporting contexts explored in this paper, including running, kettlebell training, capoeira, and surfing, joy is expressed through embodied practice and relational engagement. Whether in the rhythm of running together, the strength and discipline of kettlebell training, the collective exchange of capoeira, or the play and possibility of the ocean, these experiences reveal ways of being that centre Black life. These histories, and the ideological and practical systems that emerged from them, continue to shape social and political life, including sport and its relationship to Black and gendered histories and bodies. The relationship between fugitivity and freedom reflects the problematic discourses and social and political realities Black people experience in and through sport in everyday spaces.

Hypervigilance, for instance, reveals how Black visibility, particularly when engaging in sport in predominantly white spaces, shapes questions of safety and determines how sport is accessed and experienced (48). These linkages manifest through ideas and images of criminality that follow Black individuals, as seen in the death of Ahmaud Arbery, a young Black man who was hunted down and murdered while jogging through a predominantly white neighbourhood one afternoon in 2020 (48). They are also reflected in everyday life, where Black children playing outdoor sport in their neighbourhood can become the site of violence, as in the death of a Black mother (49), and where Black women and girls who move individually or collectively remain subject to surveillance (3). Within a shared global Black identity in sport and sporting spaces, surveillance remains both a central condition and a lived reality for Black people engaging in sport.

Ideas of freedom exist alongside the realities of fugitivity that confront erasure. Black women in sporting spaces show up in ways that refuse what fugitivity and captivity suggest about Black women's visibility, including what that visibility should and should not look like (50). Reading Black women's bodies as evidence brings attention to how ideas capture and display through movement reproduce hierarchies of power, vigilance, and the collective gaze (51, 52). These structures not only reinforce stereotypes of Black women but also shape the ways in which identity is negotiated through participation in sport. For example, Simone Biles and Naomi Osaka's prioritization of their mental health over sport participation demonstrates how Black women in sport promote care and resistance through refusal (53). It shows up through collective refusal, in which Black women gather, share in sport, and contribute to a sense of community – sport is sport a site of safety and homeplace (54, 55). While power can be understood through explicit hierarchies, photographs of Black women athletes also raise questions about how power is reclaimed or redistributed among Black women through choice and care. Black women's simultaneous refusal of fugitivity is an act of preservation, one that not only demands but commands attention to the nuanced, complex, and liberatory ways sport and global identities are shaped and shared in sport.

Colonial futures are guaranteed as the matrices of power that govern media, health, education, and sport become more and more deeply entrenched. As such, refusals to submit and finding ways to bolster ideas and experiences of fugitivity and freedom will be ever more important to guarantee a futurity against capture. Campt (9) insists that Black feminist futurity relates to the possibility of being demanding, dogged and disruptive. A politics of refusal centers on the rejection of the disappearing nature of white supremacy, rendering Black people, particularly Black women, as “fundamentally illegible and unintelligible,” as ‘other’ or ‘object’ [(56),para. 19 (57, 58);] In doing so, this frame resists the “unrelenting pathologizing” of Black people within the global context of anti-Blackness [(59), p. 1194]. To consider what we will be in the future, we must consider what are we refusing today? The images above position refusal through sport not as stopping, shutting down, or turning away. Rather, the runner, surfer, kettlebell lifter, and capoeira players engage refusal as everyday actions. Beyond protest and conventional resistance models, it is the quotidian images of Black subjects whose daily efforts are frequently overlooked that can help us to understand refusal (9). The efforts of athletes to engage in their daily practice, to be healthy, centered in community, or one with the sea and land, as the above images reveal, are examples of quiet photography, demonstrating the imaginatory self (and family, community, and future generations) that thrives despite ongoing anti-Black racism and misogyny. The runner or surfer to goes out on the land/sea for themselves; they do not do it for the cameras. The capoeira players in the studio and the kettlebell lifter in the gym do it for the tension and release in their bodies. They engage in sport as an active refusal of erasure, striving for Black futures.

## Ritual and care

In this paper, ritual is understood as multifaceted. It is both a personal and social practice that functions as a site of refusal by reclaiming and retaining agency while navigating overlapping experiences of racism and sexism within and beyond sport spaces. Ritual allows Black girls and women to name and experience joy as a tool for surviving and thriving in oppressive and exclusionary environments. Dating back centuries, Finch (60) describes how enslaved women engaged in audacious living that produced rival geographies, defiant forms of kinship, and organized refusals of slavery, naming this “violence-breaking” as an early form of care and refusal that laid the groundwork for twentieth-century activism (p. 10). Alongside the realities and experiences of joy, there remains a critical underpinning of systemic, state, and colonial violence, as well as punitive geographies shaped by war and occupation, that inform sporting spaces in the afterlife of slavery (60). The consequences and harms, as Finch (60) argues, “take up residence in the body, erode the life force, produce cellular wounds, and beckon literal death,” all of which require and center radical forms of care. As such, Lorde's (61) assertion that self-care is “political warfare and an act of survival” becomes a reverberating ritual that necessitates revisiting, integrating, and imagining anew, requiring that we move beyond simplistic assessments of care to understand its complex historical implications for Black girls’ and women's experiences in the twenty-first century.

Sport sociologists for over 50 years have detailed the ways sport operates as a ritual, ceremony, and even quasi-religion. Birrell (62) draws from Émile Durkheim and Erving Goffman to show the function of ritual in sport as generating rules of conduct in relation to “objects or values designated as special or sacred” [(62), p. 357]. Sacred running paths, beaches, gyms or studios that enable not necessarily the exciting or spectacular but daily life interactions that comprise social experience and reaffirm values – such as land sovereignty for Jamaican surfers. In addition to environmental connection, sacred and ritualistic practices are part of Black women and girls’ body reclamation through sporting practices. Self-adornment, as Black women's aesthetic practice, is an act of ritual and self-care engaged through sport and the moving body. It can be understood as both personal and political, as it warrants a commitment to self-adoration, acceptance, and determination by showing up in sporting spaces and taking up space as agentic and liberated people. The deliberate choice to dress oneself through clothing, hairstyles, jewelry, hygiene, body modifications such as tattoos and piercings, and other forms of material stylistic expression has supported self-definition for Black women (63, 64), including in sport (1, 2). Black women's adornment practices challenge white or Eurocentric aesthetics and ideals of femininity and beauty, alongside colonial stereotypes and tropes about Black women's bodies.

Cole and Zucker (65) argue that dominant ideals of femininity closely tied to white womanhood are valued within broader American culture. Chen (66) adds that these ideals are reproduced and made pervasive through systems, institutions, and social networks. This supposition narrows the ways femininity is understood. Collins (67) refers to as hegemonic femininity: a set of beliefs about desirability and gendered practices that represents a dominant measuring tool for all femininities, whereby “Black women are regulated to the bottom of the hierarchy” [(67), p. 193] and never measure up. In dominant images of sport, this rendering and regulating marks Black women's bodies as legible sites where insufficiency, deficit, or ugliness are mapped onto them. As a consequence of the narrow mapping and misguided representations, Black women's resting and moving bodies are informed by, and inform, how they are understood. Rituals of adornment and care can become that much more important, acting as both a symbolic form of resistance and reclamation in sport. For example, the presence of a Black girl surfing disrupts dominant imaginaries, asserting not only belonging, but a deeper cultural and historical relationship with the ocean and with one another. Building on Hylton and Munroe (68) and their interdisciplinary project exploring Black girl surfers in Jamaica, care is understood as relational and embodied, even within shared cultural contexts. This requires vulnerability, expressed through what Henry (69) describes as a practice of listening as care. Hylton and Munroe (68) demonstrate this through their participation in the ocean, where they engage in shared processes and rituals of learning, failing, laughing, and trying again. While acts of adornment and care are influenced by geographic location, cultural traditions, and personal choice, there is a shared global sameness in confronting and defying the politics of respectability, hypervisibility, and sexualization.

Defining Black womanhood and identity through sport and movement is a complex and critical praxis for Black women. In everyday ways, whether as high-performing athletes or through recreational sport in community settings, self-adornment as a ritualistic practice moves beyond physical descriptors in sport, such as strength, resilience, and body awareness, toward significant social implications for how Black women make sense of themselves. Tillet and Tillet's (70) work on Black girls, paired with our inclusion of images, challenges traditional conceptions of what art is and how it is used, moving beyond art education as simply looking at images and toward looking with them (10, 70). In looking back, art takes on new meaning, and so too do aesthetics and identity. In performance, sport, and the moving body, Black women display an aesthetic that carries histories of movement and migration, care, tradition, stories, and shared identity. Aesthetics becomes not only a vessel for informing practices around beauty, femininity, and identity, but also a ritualistic practice of adornment: a preparation for, and a way of being through, the moving or sporting body. In this way, adornment represents a means by which Black women understand themselves and are shaped by themselves, their histories, and the spaces they move through. In every sport, the equipment, artefacts, materials, symbols, adornments, and movements connect traditions of the past with the possibilities of today. Joy is not optional or incidental in these rituals (34). It is vital to sustain collective well-being and Black flourishing.

In sport, rituals are represented through repetition, reinforcing memory, community, and embodied commitments such as physical strength, mind-body awareness, chants, cheers, and preparatory practices. Manning (71) discusses rituals of incorporation, which focus on bringing new members into a culture, as well as rituals of entering and exiting, which shape how events or activities begin and end, mark the transition from outsider to insider, and define membership, identity, and belonging. Rituals are generative, as they reflect the norms, values, and expectations of a group while also creating a sense of belonging and identity. As Collins and Lewis (72) note, rituals provide “meaning and relevance to institutions while at the same time connecting with the past” (p. 47), linking present participation to shared histories and collective memory. While ritual practices may shift over time through the integration of new ways of doing and the participation of different generations of women, their foundations remain through shared symbolic acts and materials. Through ritual, structure and ceremony are created, becoming central to performance and belonging, as they help athletes understand who they are within a collective. In each context of the images presented above, movement becomes ceremony, an embodied form of knowledge that is both personal and collective, shared communally and carried across generations. These ritual practices point to broader ways of understanding how Black women and girls create meaning and possibility, contributing to a shared global Black identity across sporting contexts and beyond.

## Joy is in our movement; community is how we care

Building on the possibilities of refusal and ritual, this section brings together the central themes of the paper, highlighting how Black women and girls thrive through practices of community care, joy, and collective knowledge production. Despite ongoing forms of racial and gendered oppression, Black women continue to cultivate spaces of collective joy that are generative (56, 73, 74). These practices are forged through necessity, emerging from conditions in which traditional pathways to participation, movement, expression, and connection have often been denied. As a result, Black women and girls develop creative and nontraditional ways of engaging in physical activity, whether through running, strength training, or other forms of movement that exist outside dominant, exclusionary models (75–77). These practices of joy are ritualistic in that is more than sports; these places are sanctuaries. These rituals produce alternative modes of participation but also sites of joy, connection, and world making (78).

Joy, whether explicitly named or not, makes visible forms of Black presence that exist beyond dominant narratives of exclusion and are inseparable from refusal (34). Joy operates not only as a feeling, but as a practice. It challenges anti-Blackness and gendered assumptions that position Black women and girls outside of girlhood, womanhood, and femininity. Within, these practices assert Black women and girls are position as belonging fully within sporting spaces and beyond them. Moments of preparation, exchange, and movement across other sporting contexts illustrate how joy and connection emerge through shared participation take shape and extend beyond formal play. Community is central to these experiences. Through sport, Black women and girls build relationships that hold and sustain them, creating spaces of care, safety, and possibility (68). These communities are not incidental; they are actively produced through practices of gathering, supporting, and moving together illuminating relationships to self and the collective that remain central aspects of these experiences. Care circulates within these spaces through mentorship, storytelling, and collective presence. In this way, community and care become foundational to how Black women and girls engage sport.

Resisting the conditions that seek to constrain Black life by affirming presence, embodiment, and possibility, joy and community are not only oppositional but generative. It enables Black women and girls to create alternative ways of being within and beyond sporting spaces challenging the idea that parts of oneself must be left behind or that Black women “don't do that”. Instead, they affirm that Black women and girls move through sport with their full identities, histories, and subjectivities intact. These practices can also be understood as ritual, as repeated, embodied actions that sustain connection across time and space. Sporting spaces become more than sites of activity; they become spaces of meaning, where relationships are built, memories are formed, and collective futures are imagined. Through these practices, Black women and girls create spaces that are not only participatory but sustaining.

It is through this creativity that new forms of knowledge are produced, grounded in Black girls’ lived experience and imbued with relationality and care (60, 79, 80). This capacity for joy thereby functions as a form of resistance (73). The creation and sustenance of joy, too, is part of a politic of refusal, contesting dominant narratives that focus on pain, suffering, and marginalization (81, 82). In this way, collective joy is not separate from resistance it is one of its most vital expressions, enabling Black women and girls to thrive while simultaneously challenging the structures that seek to constrain them.

### Prelude to conclude

#### Joy and cares live here

Not as an afterthought, but as practice. As presence. As something built together. And with it, community follows. It holds. It sustains. It makes space for breath, for learning, for being. Care moves through these moments, quietly and persistently, in the ways Black women and girls support, witness, and remain with one another. Together, these practices of joy, community, care, and refusal reveal how Black women and girls generate knowledge through lived experience and relational engagement. These forms of knowledge are grounded in Black feminist epistemologies, where embodiment, connection, and relationality are central. Joy and community are actively produced through these practices, shaping how Black women and girls live, move, and imagine within and beyond sport. Across many global contexts, while experiences differ, Black women and girls remain connected through shared histories of colonialism and ongoing structures of inequality. Within these conditions, Black women and girls continue to refuse, ritualize, and cultivate joy, sustaining themselves and one another while creating new possibilities for Black life. When Black women take up space in sports that have often excluded them, their rituals of care, healing, and celebration of motion become tools of socialization and acts of resistance.

They gather in motion.

In the rhythm of running beside one another.

In the preparation before a kettlebell swing.

In the balance and drift of the ocean surf.

In the exchange and timing of capoeira kicks and escapes.

## Conclusion

Our gathering, writing, and analysis of these four images demonstrate how this approach can excavate and elicit new meanings about ourselves and the global identities of Black women in sport. Engaging in interdisciplinary, reflective, arts-based, and collaborative storytelling, we collectively reflect on our individual experiences across distinct research studies while recognizing personal narratives and the body as crucial sources of knowledge and memory. Although our personal histories and lineages to each of the four countries differ, they each share a history of colonization.

Centring the experiences of Black women and the reflections of Black women scholars who draw on various forms of critical thought, this work invites a dialogue that considers how Black women have shaped and continue to shape the landscape of sport. It also challenges how identity is often predicated on linear, narrow understandings that obscure Black women's influence and the space they take up in sport. In this context, refusal names the acts through which Black women resist containment, erasure, and marginalization, asserting presence and self-definition within and beyond sport. Ritual refers to repeated embodied practices and engagements in movement, gathering, memory, and expression that sustain connection across time and space.

The photos from four areas of the world, portraying Black women indoors and outdoors, as part of groups and as individuals, on land and at sea, reveal the richness of Black cultural diversity. The feelings evoked through this analysis are of our similarities across difference and a deep knowledge of cultures that are not singular but interconnected through shared histories of anti-Blackness and refusal to be contained, eliminated, or weakened. In sport, Black women and girls rise above and against these conditions, engaging in both refusal and ritual in ways that situate them firmly at the centre rather than the margins of sport. Identity, then, contributes to a shared global understanding of Blackness, shaping a culture and aesthetic of refusal expressed through personal style, dress, and embodied practice. These expressions bring together ritual practices with forms of joy and collective care. This paper works to provide new insights into exploring sport and identity broadly, and particularly as they relate to Black girls and women.

We acknowledge that the audience for this article, and the athletes featured in the photographs may have other interpretations that could deepen these findings. Furthermore, these athletes and others, operating in different sports and different geographies, could lead to alternative visual stories. We welcome opportunities for future research and community dialogues. We move beyond high-performance sporting spaces to everyday, ordinary environments to engage the ways in which sport shapes, is shaped by, and contributes to Black identities, globally and more locally.

## Data Availability

The original contributions presented in the study are included in the article/Supplementary Material, further inquiries can be directed to the corresponding author.
